# Medical and Hospital Progress at Oxford

**Published:** 1894-12-15

**Authors:** 


### The Hospital

An Institutional Journal of

The Medical Sciences and Hospital Administration, with

AN EXTRA NURSING SUPPLEMENT.

Vol. XVII.—No. 429.] [Dec. 15, 1894.

### Medical and Hospital Progress at Oxford.

It is more than a satisfaction to the medical lovers of Oxford, and they are many, to see grounds for hope that the Radcliffe Infirmary, and with it concrete medicine, is at last moving forward at our greatest University. Medical men, as well as others, recognise the part Oxford has played in the advancement of the highest culture among English-speaking peoples throughout the world during the last six or seven centuries. For learning in general, and for theological learning in especial, Oxford has done yeoman service during many ages. What she has done for theological and general learning it is now desired by all cultured medical souls she shall do for medicine. Not only so, but if Oxford will come to the help of those whose one aim it is to make of medicine a true "science of man," great will be the services she will render to the world, and greater will be her modern than even her ancient glory.

But if Oxford is to play this great part the first of all conditions is that she be practical. To be practical, she must begin at home, and must not "despise the day of small things." Oxford has a medical school, and she has a hospital. But what is her medical school, what can it ever be, unless her hospital be the home of all the sciences of man and medicine, and of all those sciences at their best? Is the Radcliffe Infirmary at the present moment the home of all the sciences of man and medicine at their best? Has Oxford—Oxford as the world understands it, the Oxford of the University—ever made a serious effort to raise the Radcliffe Infirmary to the level of a home of the highest science of medicine and of man? We put these questions to academic Oxford; and that Oxford knows, without so much as the shadow of a suggestion from us, exactly how they should one and all be answered.

If we must be plain, we are compelled to affirm at once that the Radcliffe Infirmary is not in any high sense a "home of science" at all. It is a provincial infirmary, managed on provincial lines, and does not even profess to give any leadership of any kind to the medicine of Oxford, or the University, or the country. Is this the position a hospital should hold which is the hospital of a medical school? Above all, is it the position a hospital should hold which is the hospital of the medical school of the University of Oxford?

The people of Oxford have mended their infirmary in certain important particulars, and that is well. But that task was one for mere craftsmen compared with the real duty which lies before the infirmary managers and the heads of the University. The task which now lies before those at Oxford who are capable of statesmanship is the reconstruction, not of the infirmary buildings only, but of the medical school, the nursing department, and every vital portion which helps to constitute the complete organisation of the hospital. What is wanted at Oxford is an institution, small it may be as to the number of its beds, but in every essential particular of structure, of management, and of high scientific aims second to none in the whole world as a hospital and medical school. This, and nothing less than this, should satisfy the managers of the Radcliffe Infirmary and the heads of our greatest University.

To come a little more to details. The internal management of the infirmary is not modern, it is not vitalised by the modern spirit. How is its vitalisation to be effected? The modern spirit must be taken into the hospital in a modern body. A competent, cultured, liberal minded, medical superintendent is needed who can exercise efficient control over every department of the institution. It will not be affirmed by any person who knows the inner daily life of the Radcliffe Infirmary that medical science, in its modern sense, is the ruling, controlling, inspiring spirit of the place. How can it be, unless there be a medical man or medical men in and about the institution every hour of the day endowed with knowledge and the modern spirit, and in whose hands can be placed the key of authority and control? It is medical and scientific vitality which is wanted, and which can alone be found in the person of a competent medical and scientific superintendent of the necessary age, status, and authority. Such a person is found needful for Guy's and St. Thomas's. Such a person is imperative at Edinburgh and other large hospital medical schools combined with and inspired by Universities. Such a person, if he were but once found, would impel new life into every part of Oxford's medical activity.

But the secret of secrets of the languor and backwardness of the Radcliffe Infirmary is its practical severance from the life and activity of the University. The University, instead of being the tender mother of the most human of all the sciences, the science of medicine, does not care to admit even that she is a stepmother. If we are to believe the *Oxford Journal* she takes no interest of any kind in the infirmary, and gives no practical help, not even the poor help of a money contribution. It is true that individual members of the University, such as Dr. Bright and others, have from time to time devoted both energy and money to the work of the infirmary; but the University as such has, like the Priest and the Levite in the parable, ostentatiously gathered up her skirts and "passed by on the other side."

To our thinking this is all very pitiful. Whatever medicine may have been in the past she is now a noble gladiator striving in the arena for the highest physical amelioration of the lot of man. Is not this an object worthy of the learning and culture of any age or generation, and is it not seen in its noblest form in the daily activity of a hospital? If Oxford is ashamed of association with her hospital she has studied to little purpose either the sciences on which modern medicine is based, or the ancient literatures and theologies in which the love of humanity occupies so conspicuous a place. Our Special Commissioner has suggested that the Radcliffe Infirmary should realise £5,000 of her invested capital in order to give herself a new start on modern lines. Let her do so; expending that or even more for the purpose; let her build herself a home worthy of medicine and of medical science; let the University spread forth its ample wing and protect and nourish the struggling institution; let all Oxford of every class join in the great work, and let the Radcliffe Infirmary become in the future conspicuous for all that is great in medical science —a school and a hospital worthy of so famous a seat of learning.

